# Transarterial embolization in musculoskeletal care: an indication-stratified narrative review

**DOI:** 10.1186/s42155-026-00776-7

**Published:** 2026-10-03

**Authors:** Jean-Pierre Pelage, Thunayan Aldalmani, Ruben Geevarghese, Norbert Zhang, Marc-Antoine Jegonday, Gregory Berry, Anthony Dohan, Audrey Fohlen, Sylvain Grange

**Affiliations:** 1 Department of Diagnostic Radiology, McGill University Health Centre (MUHC-Glen)-Royal Victoria Hospital, Montreal, Canada; 2 Department of Diagnostic Imaging and Interventional Radiology, CHU de Caen, Normandie University, Avenue de La Côte de Nacre, Caen Cedex 9, 14033 France; 3 Division of Interventional Radiology, Department of Radiology, Memorial Sloan Kettering Cancer Center, 1275 York Avenue, New York, NY USA; 4 DMU Smart Imaging, Medical Imaging and Interventional Radiology Department, Assistance Publique-Hôpitaux de Paris, GH Université Paris-Saclay, Ambroise Paré Hospital, Boulogne-Billancourt, France; 5 Department of Radiology, CHU de Rennes, Rennes University, Rennes, France; 6 Division of Orthopaedic Surgery, McGill University Health Centre, Montreal, QC Canada; 7 Department of Radiology, Hôpital Cochin, Assistance Publique-Hôpitaux de Paris, Paris, 75014 France; 8 Universite Paris Cite, CNRS, INSERM, Institut Cochin, Paris, France; 9 Department of Radiology, Radiation Oncology and Nuclear Medicine, Université de Montréal, Montreal, QC H3T 1J4 Canada; 10 Laboratoire Clinique de Traitement de L’image (LCTI), Centre de Recherche du Centre Hospitalier de L’, Université de Montréal (CRCHUM), Montreal, QC H2X 0A9 Canada; 11 Université Jean Monnet, CHU Saint-Etienne, Service de Radiologie, Mines Saint-Etienne, INSERM, SAINBIOSE U1059, Saint-Etienne, France

**Keywords:** Musculoskeletal embolization, Transarterial embolization, Trauma embolization, Genicular artery embolization, Knee osteoarthritis, Tendinopathy, Adhesive capsulitis, Recurrent hemarthrosis

## Abstract

**Background:**

Transarterial embolization (TAE) is increasingly used across a broad spectrum of musculoskeletal conditions, ranging from established hemostatic and tumor-related indications to emerging pain-directed procedures for degenerative joint disease and chronic inflammatory tendinopathies. Because these applications differ substantially in clinical maturity, methodological quality, durability of outcomes, and comparative evidence, an indication-stratified synthesis is needed.

**Main body:**

This narrative review summarizes technical principles, vascular anatomy considerations, embolic agent selection, clinical indications, and evidence maturity in musculoskeletal TAE. Hemostatic and selected tumor-related applications are established components of multidisciplinary care, whereas pain-directed embolization, including Genicular artery embolization for knee osteoarthritis and embolization for tendinopathies, is generally performed as a stand-alone minimally invasive procedure targeting pathological neovascularity. Current evidence remains heterogeneous, with variable study designs, follow-up, endpoints, and definitions of technical and clinical success.

**Conclusions:**

Musculoskeletal TAE is an expanding field with established, intermediate, and exploratory indications. Its evidence base is strongest for hemostatic and selected tumor-related applications, whereas stand-alone pain-directed embolization requires careful patient selection, standardized outcome assessment, and further comparative trials to define durability and clinical value.

## Highlights

- Musculoskeletal embolization is a rapidly evolving field in interventional radiology.
- Its indications now extend beyond bleeding to tumors and chronic musculoskeletal pain.
- Procedural success depends on precise vascular mapping and careful patient selection.
- Embolic materials should be selected according to the anatomy and clinical objective.

## Introduction

Transcatheter arterial embolization (TAE) involves the deliberate occlusion of selected blood vessels to control hemorrhage or treat conditions such as hypervascular tumors by delivering embolic agents through a catheter-based procedure. Since its first reported use in 1972 in the management of pelvic hemorrhage [1] and life-threatening pelvic trauma [2], the role of TAE has progressively expanded to include the treatment of hypervascular tumors, postoperative complications such as recurrent hemarthrosis, and inflammatory or degenerative arthropathies. Pre-operative embolization of hypervascular bone tumors can effectively achieve tumor devascularization and significantly reduce intraoperative blood loss [3]. Spontaneous or postoperative recurrent hemarthrosis, particularly in patients with hemophilia, may also be successfully treated with TAE [4]. In addition, patients presenting with intractable pain related to bone lesions may benefit from embolization as a palliative strategy to reduce pain [5]. Abnormal neovascularization, along with associated nerve ingrowth, is believed to play a key role in the pain seen in chronic inflammatory and degenerative arthropathies [6]. As a result, TAE has emerged as a potential therapeutic option for chronic tendinopathies and has been evaluated across multiple anatomical sites [7].

Musculoskeletal TAE encompasses six broad applications: emergency and postoperative hemostasis; tumor-related embolization; adjunctive embolization before or during percutaneous treatment; vascular malformations and aneurysmal bone cysts; recurrent hemarthrosis; and stand-alone pain-directed embolization for osteoarthritis and tendinopathies. Existing reviews often discuss these applications together despite major differences in therapeutic intent, evidence quality, follow-up, and clinical maturity. This narrative review therefore provides an indication-stratified synthesis, distinguishing established hemostatic and selected tumor-related uses from less mature pain-directed procedures.

## Methods

### Literature search and article selection

This article was designed as a narrative review rather than a systematic review or meta-analysis. A structured literature search was performed in PubMed/MEDLINE, Embase, Scopus, and Google Scholar, with the final search conducted on June 24, 2026. Search concepts combined embolization-related terms with indication-specific terms covering trauma and postoperative hemorrhage, bone and soft-tissue tumors, aneurysmal bone cysts, vascular malformations, hemarthrosis, osteoarthritis, tendinopathies, adhesive capsulitis, and other musculoskeletal applications. Reference lists of selected articles and major reviews were also screened.

English-language clinical studies, systematic reviews, narrative reviews, technical reports, and consensus or guideline documents were considered. Case reports and small case series were included for rare or emerging indications when higher-level evidence was unavailable. Article selection was performed collaboratively by the authors rather than through formal duplicate independent screening, and disagreements were resolved by discussion and consensus.

Consistent with the narrative-review design, no formal PRISMA-based study-selection process, risk-of-bias assessment, or quantitative pooling was performed. Evidence maturity was characterized qualitatively according to study design, sample size, comparative evidence, consistency of reported outcomes, follow-up duration, safety data, and current clinical use. These categories were developed by the authors specifically for this narrative review and do not represent a formally validated evidence-grading system or a guideline/consensus classification. Because study identification and selection were not conducted as a formal systematic screening process, we did not report an approximate included-study count, as this could imply a degree of systematic exhaustiveness that was not intended for this narrative review.

## Vascular anatomy of major joints: key considerations for embolization

**Fig. 1:**
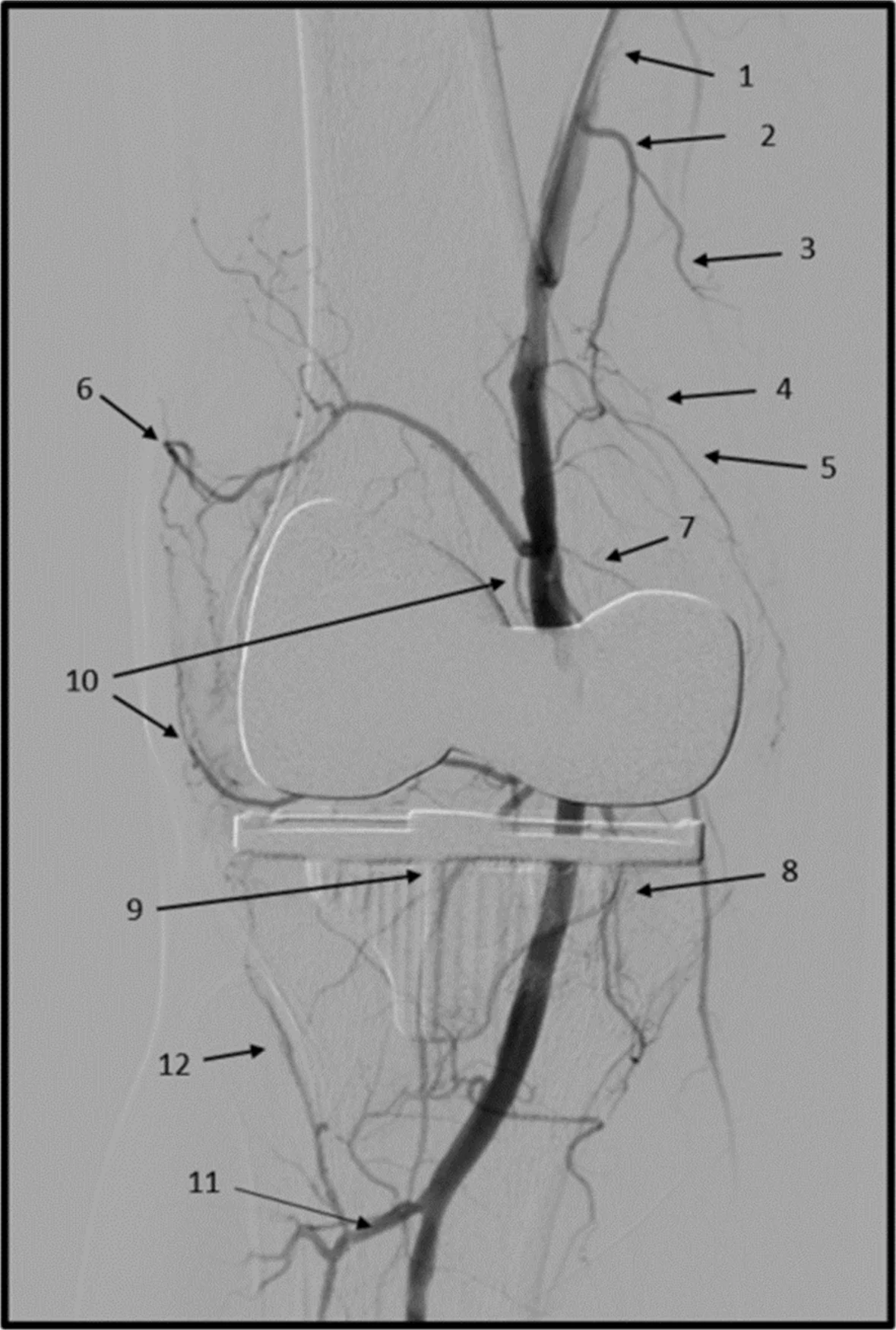
Knee vascular anatomy on frontal arteriography (arterial phase). (1) Popliteal artery (PA). (2) Descending genicular artery (DGA). (3) Saphenous branch of the descending genicular artery (SB). (4) Muscular branch of the descending genicular artery (MB). (5) Osteoarticular branch of the descending genicular artery (OAB). (6) Superolateral genicular artery (SLGA). (7) Superior medial genicular artery (SMGA). (8) Inferior medial genicular artery (IMGA). (9) Inferior lateral genicular artery (ILGA). (10) Middle genicular artery (MGA). (11) Anterior tibial artery (ATA). (12) Anterior tibial recurrent artery (ATRA)

A thorough understanding of MSK vascular anatomy is essential for the safe and effective performance of TAE. This includes knowledge of arterial origins, branching patterns, collateral pathways, and potential cutaneous, muscular, or neural branches at risk of nontarget embolization. The knee is discussed in greater detail because Genicular artery embolization is the most extensively studied stand-alone pain-directed musculoskeletal embolization procedure and because genicular arterial anatomy is complex [8], variable, and directly relevant to both efficacy and safety (Fig. 1). Other territories are summarized more concisely to preserve the focus of this narrative review. The elbow is supplied by a dense periarticular collateral network connecting branches of the brachial, profunda brachii, radial, ulnar, and interosseous arterial systems. Relevant contributors include the radial collateral and middle collateral arteries, the superior and inferior ulnar collateral arteries, and the radial, ulnar, and interosseous recurrent arteries. In lateral epicondylitis, pathological hypervascularity is typically sought around the common extensor origin. Superselective catheterization and careful angiographic assessment are required to target abnormal neovessels while avoiding nontarget embolization of muscular, cutaneous, or neural branches [8].

## Embolic agents (Table 1)

**Table 1:** Embolic agents used in musculoskeletal transarterial embolization. This table summarizes the main embolic agents used in musculoskeletal transarterial embolization, including their usual characteristics, common clinical indications, advantages, and limitations. Agent selection should be individualized according to the therapeutic objective, target-vessel caliber, desired level and duration of occlusion, collateral supply, bleeding pattern, and risk of nontarget embolization

Agent/class	Typical size/characteristics	Common MSK indications	Advantages	Main limitations
Gelatin sponge	Temporary; pledgets/slurry; variable particle size	Trauma or post-operative bleeding; selected temporary devascularization	Widely available; temporary; useful in acute bleeding	Variable particle size; recanalization; less suited when durable distal embolization is required
PVA particles	Commonly 100–300, 300–500, or larger micrometer ranges, depending on indication	Tumor devascularization; hemarthrosis; selected hypervascular lesions	Permanent distal embolization; widely used	Particle aggregation; less calibrated than microspheres; non-target risk
Calibrated microspheres	Calibrated particles; size selected according to target vessels	Tumor-related embolization; hemarthrosis; selected inflammatory targets	More predictable size distribution	Permanent; cost; non-target ischemia if too small or poorly selected
Imipenem/cilastatin slurry	Small temporary crystalline particles; preparation-dependent	GAE and selected pain-directed embolization procedures	Temporary distal embolization; frequently reported in GAE literature	Off-label in many settings; preparation variability; availability/regulatory considerations
Iodized oil	Radiopaque oily agent; may be used alone or in mixtures according to protocol	Selected pain-directed or tumor-related procedures in published protocols	Visibility; distal penetration	Technique-dependent; evidence remains indication-specific
Coils/plugs	Mechanical proximal or focal occlusion devices	Pseudoaneurysm, focal arterial injury, arteriovenous fistula, selective hemostasis	Precise deployment; durable occlusion	Not appropriate for diffuse pathological blush; collateral recruitment; requires adequate landing zone
NBCA	Liquid adhesive embolic; mixed with iodized oil	High-flow bleeding; pseudoaneurysm; selected vascular malformations/tumors	Rapid durable occlusion; useful in coagulopathy	Requires expertise; catheter entrapment; non-target embolization risk
EVOH-based liquid embolics	Non-adhesive liquid embolic	Selected vascular malformations or complex lesions	Controlled injection; durable embolization	Cost; artifact; requires expertise; longer injection time

*Abbreviations*: *ABC* aneurysmal bone cyst, *EVOH* ethylene–vinyl alcohol copolymer, *GAE* genicular artery embolization, *NBCA* n-butyl cyanoacrylate, *OA* osteoarthritis, *PVA* polyvinyl alcohol, *TAE* transarterial embolization, *TMJ* temporomandibular joint

Embolic-agent selection should be tailored to the indication, target-vessel caliber, desired level and duration of occlusion, collateral circulation, and risk of nontarget embolization (Table 1) [9–19]. Temporary agents, including gelatin sponge, imipenem/cilastatin slurry, and Lipiodol-based approaches, may be preferred when transient reduction of pathological hyperemia is intended, particularly in selected pain-directed indications. Permanent particles, coils, plugs, NBCA, or EVOH are more appropriate when durable occlusion is required, such as in active bleeding, pseudoaneurysm treatment, tumor devascularization, or vascular malformations. Because embolic materials are not interchangeable, future studies should report agent type, size, dilution, embolization endpoint, and safety outcomes in a standardized manner.

## Embolization for hemostasis

Hemostatic TAE is an established component of musculoskeletal trauma and postoperative bleeding management. It is used in pelvic ring injuries, particularly in hemodynamically unstable patients, periarticular hemorrhage associated with long-bone fractures, and postoperative bleeding around major joints. Embolization should be integrated into multidisciplinary care pathways combining resuscitation, correction of coagulopathy, imaging triage, surgery when required, and postprocedural monitoring. Contemporary management should be driven by hemodynamic status, bleeding site, associated injuries, local expertise, and the expected speed of hemorrhage control. The goal is not only technical vessel occlusion but also rapid clinical stabilization while preserving tissue perfusion through superselective embolization whenever feasible. Agent selection depends on bleeding pattern, vessel caliber, coagulation status, and the need for temporary versus durable occlusion; this is summarized in Table 1.

### Acute hemorrhage in pelvic ring and periarticular trauma

Pelvic ring injuries, usually resulting from high-energy trauma, are associated with high morbidity and mortality due to massive hemorrhage [2, 20]. Exsanguination from intrapelvic bleeding is a leading cause of early death [21]. Initial management includes application of a pelvic binder or external fixation to control venous sources, followed by angiography and embolization to address arterial hemorrhage. Arterial bleeding is identified in approximately 5–20% of cases—more frequently in hemodynamically unstable patients—and embolization achieves technical success rates ranging from 81 to 100% [22, 23].

Current management protocols emphasize contrast-enhanced whole-body CT in hemodynamically stable or transient responder patients to identify active extravasation, pseudoaneurysms, or potential target vessels [24]. In hemodynamically unstable patients with suspected pelvic arterial hemorrhage, angiography and embolization should be rapidly integrated into the center’s trauma protocol; their sequence relative to surgery, preperitoneal packing, resuscitative endovascular balloon occlusion of the aorta, or other hemorrhage-control measures depends on the bleeding pattern, associated injuries, and local resources.

**Fig. 2:**
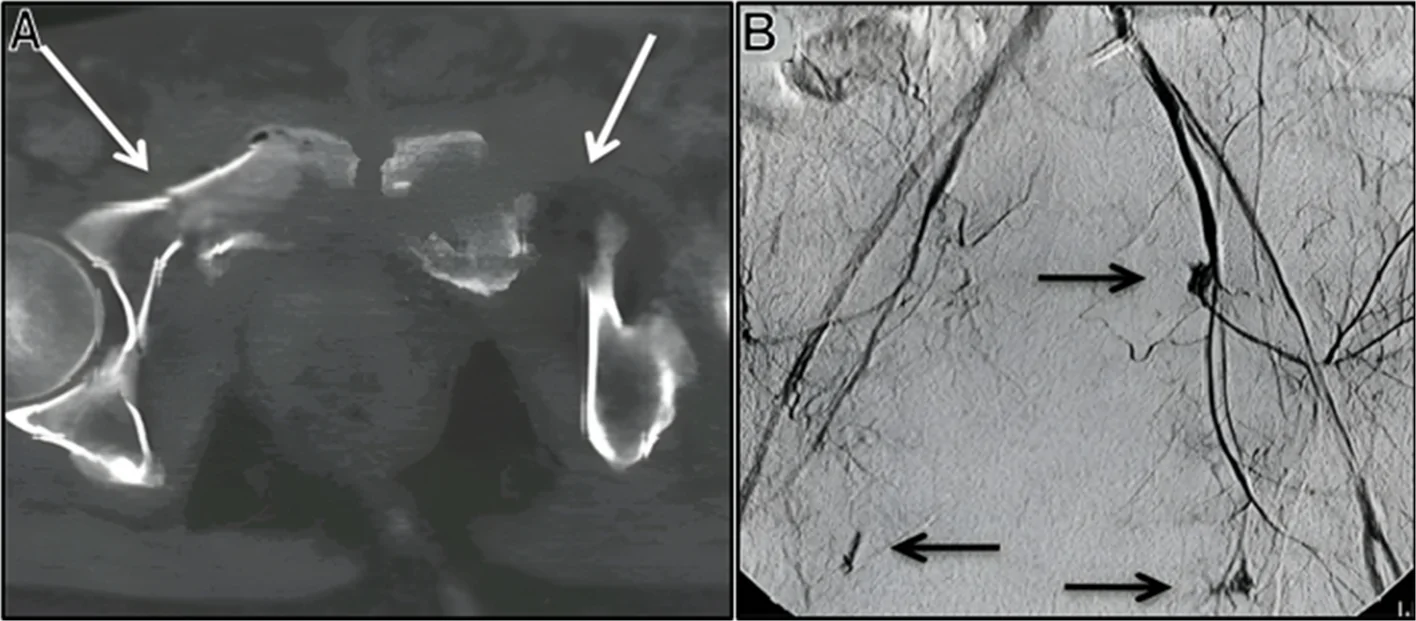
Pelvic trauma and internal iliac artery embolization. A 55-year-old man who fell from scaffolding, presenting with hemorrhagic shock and coagulopathy. **A** Pelvic CT (axial MIP). It shows bilateral ischiopubic ramus fractures (white arrows). **B** Initial frontal angiography. It demonstrates diffuse arterial vasospasm with multiple foci of contrast extravasation arising from several branches of the internal iliac arteries (black arrows)

**Fig. 3:**
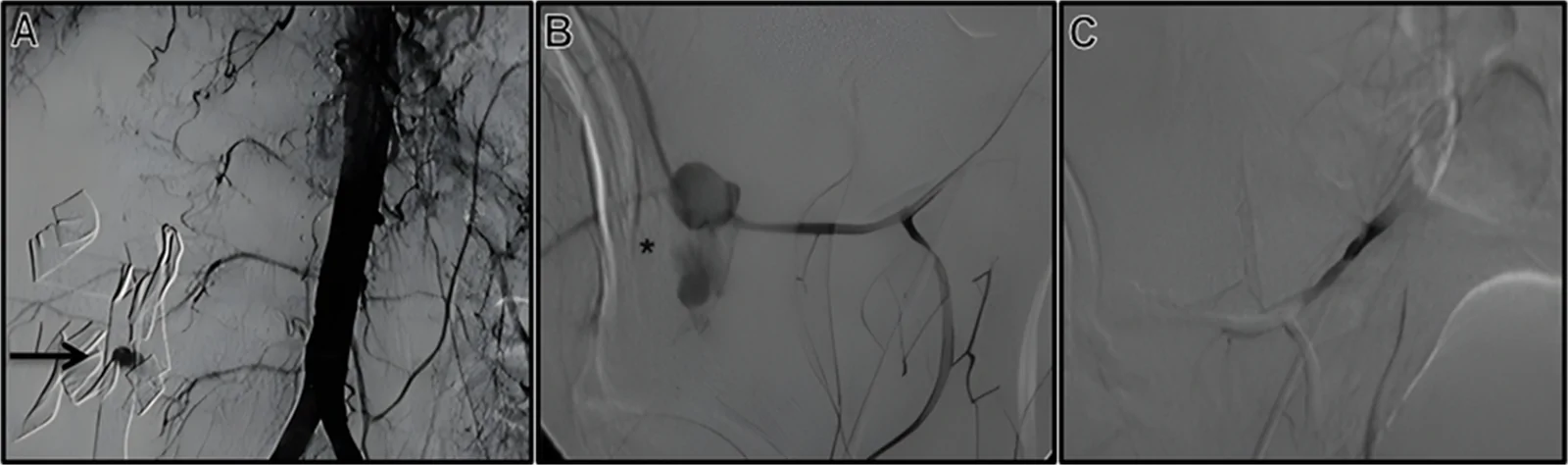
Pelvic trauma and gluteal embolization. A 27-year-old woman who fell from a height of 6 m, presenting with a large right gluteal hematoma and acute blood loss anemia. **A **Initial frontal aortography. It demonstrates extravasation of iodinated contrast material projecting over the right iliac region (arrow). **B **Selective angiography (right superior gluteal artery). It demonstrates massive contrast extravasation from the right superior gluteal artery (asterisk). **C** Postembolization selectiveangiography (right superior gluteal artery). It shows satisfactory occlusion of the target artery achieved using large gelatin sponge torpedoes

**Fig. 4:**
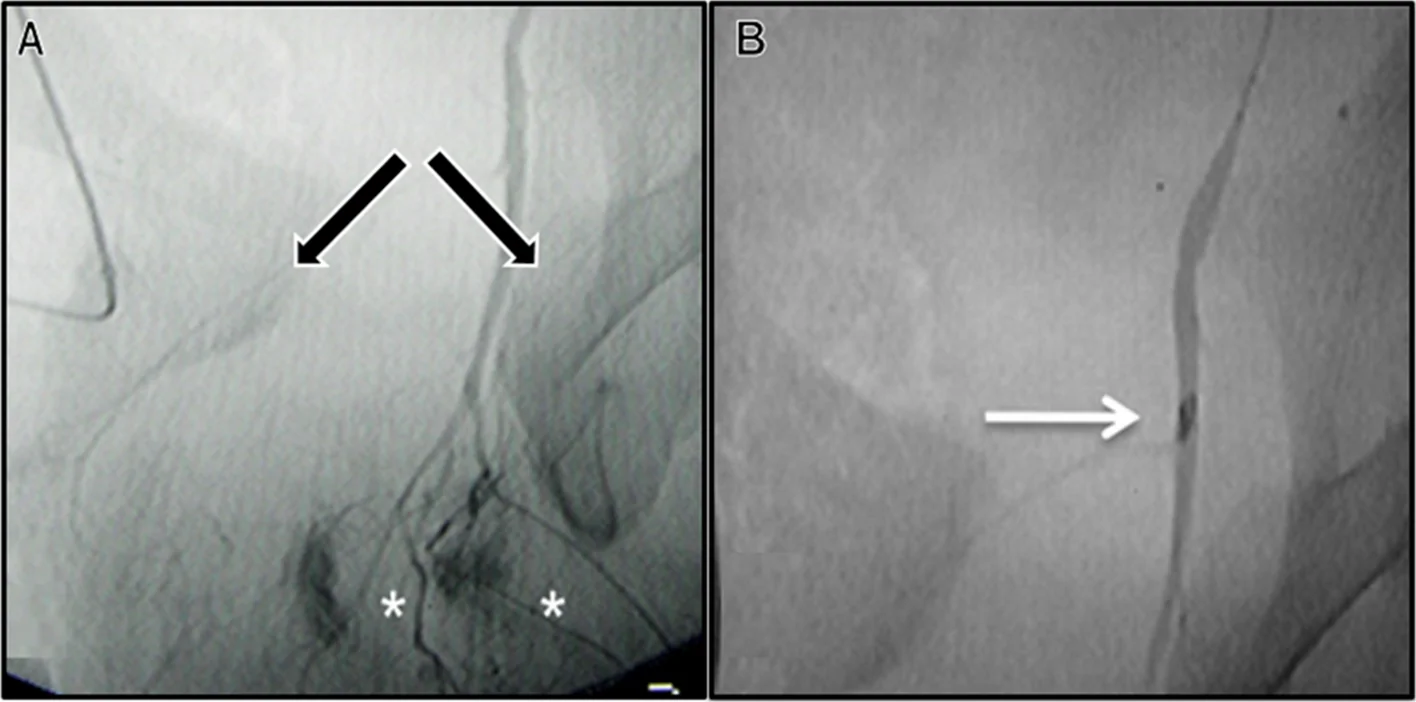
Pelvic trauma and internal pudendal artery embolization. A 39-year-old man presenting with pelvic trauma and a large scrotal hematoma after a motorcycle accident. **A **Pre-embolization frontal arteriography. It demonstrates pelvic diastasis (black arrows) and multiple foci of contrast extravasation arising from the left internal pudendal artery (asterisks). **B **Post-embolization frontal arteriography. It shows successful occlusion of the left internal pudendal artery after superselective catheterization and embolization with microcoils (white arrow)

**Fig. 5:**
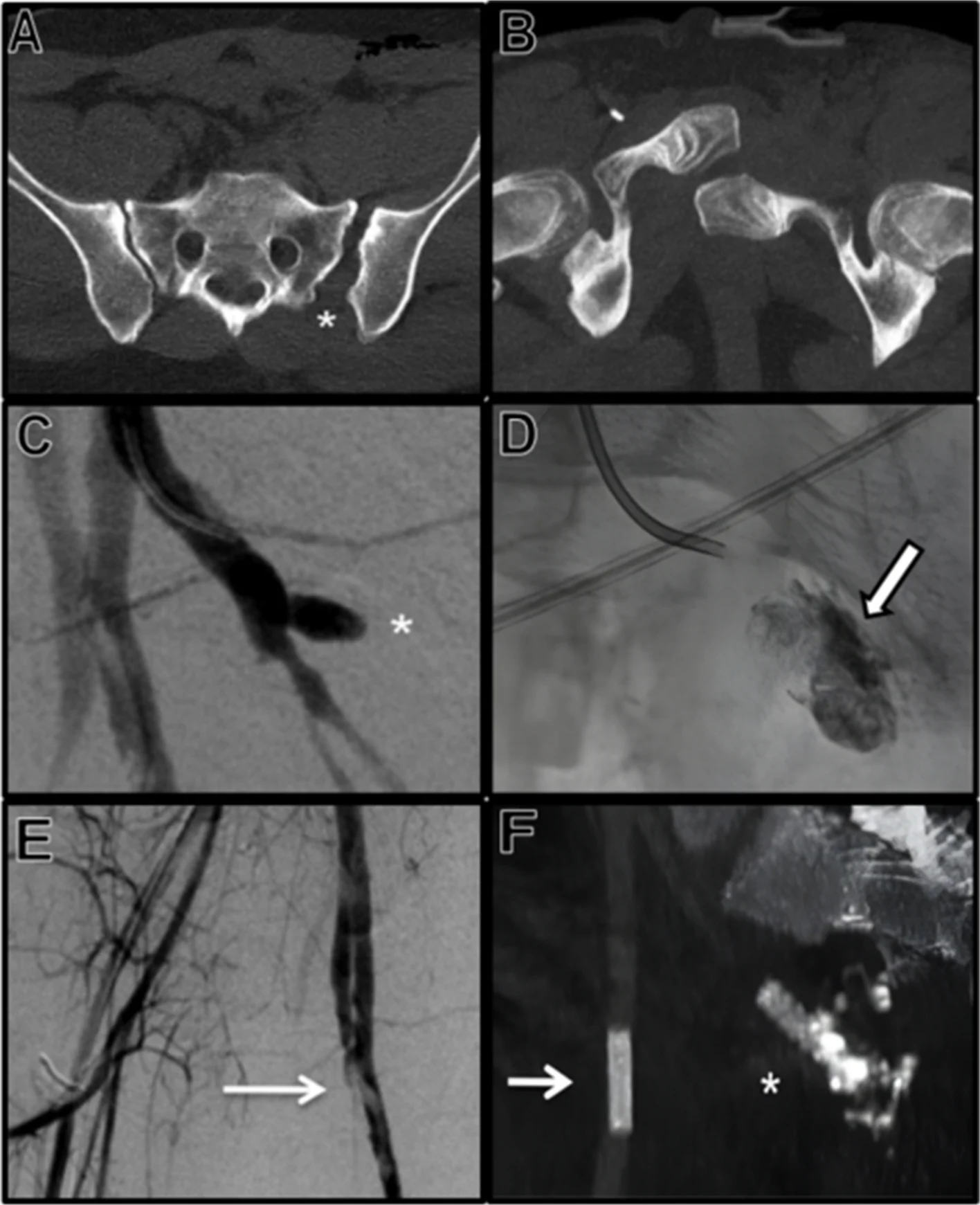
Pelvic trauma and multiple embolizations. A 25-year-old man admitted after a high-velocity road traffic accident. **A** Pre-operative CT(axial). It shows a severe left sacroiliac joint disruption (asterisk). **B** Pre-operative CT(axial MIP). It demonstrates the left sacroiliac disruption (black arrow), reflecting the severity of the trauma (severe disruption and displacement at the symphysis pubis). **C** Initial angiography (left internal iliac artery). It demonstrates a pseudoaneurysm (asterisk) arising from the distal trunk of the left internal iliac artery. **D** Angiography (left internal iliac artery). It shows superselective catheterization followed by embolization using n-butyl cyanoacrylate (NBCA) glue (white arrow). **E** Angiography (left external iliac artery). It demonstrates an arterial dissection (white arrow). **F** Postoperative pelvic angio-CT (sagittal and MIP reconstructions). It shows a covered stent placed in the left external iliac artery (white arrow) to treat the dissection. It also demonstrates persistent visibility of the NBCA glue cast (asterisk) within the left internal iliac artery

During angiography, contrast extravasation is identified in 61–100% of cases but may be absent due to spasm [25] (Fig. 2), low pressure [21], intermittent bleeding, or nonselective injection [9]. Commonly injured arteries include the superior gluteal artery (Fig. 3) and the lateral sacral arteries. The internal pudendal arteries (Fig. 4) may be injured in butterfly fractures. Injury to the internal iliac arteries (Fig. 5), as well as the obturator and inferior gluteal arteries, is typically associated with posterior dislocation of the pelvic ring (open-book fractures). The external iliac (Fig. 5) or femoral arteries may also be involved in case of anterior compression injury [9]. When a bleeding source is identified, superselective embolization is preferred. In hemodynamically unstable patients, proximal embolization using gelatin sponge may be performed to shorten procedural time [9, 21].

Resorbable gelatin sponge is the most commonly used agent. Large particles (> 700 µm) are suitable for proximal occlusion. In combined embolization strategies—often employed to achieve durable hemostasis—distal particulate embolization is performed first, followed by proximal coils to protect normal vasculature [12]. In the presence of pseudoaneurysms, “sandwich” technique, with coils placed both distal and proximal to the lesion, is recommended [9]. Liquid embolic agents, such as NBCA, may be used in selected cases, with low rebleeding rates reported [13]. Covered stent placement is less frequently performed in MSK trauma but may be considered in selected vascular injuries (Fig. 5).

Reported rates of recurrent bleeding range from 0 to 19%, with identified risk factors including initial nonselective embolization, massive transfusion requirements, and multivessel arterial involvement [23, 26]. Repeat embolization may be considered depending on the patient’s clinical status [25]. Complications related to embolization, such as skin necrosis or nerve injury, are uncommon when selective techniques are employed [21, 27].

### Embolization for postoperative hemorrhage

**Fig. 6:**
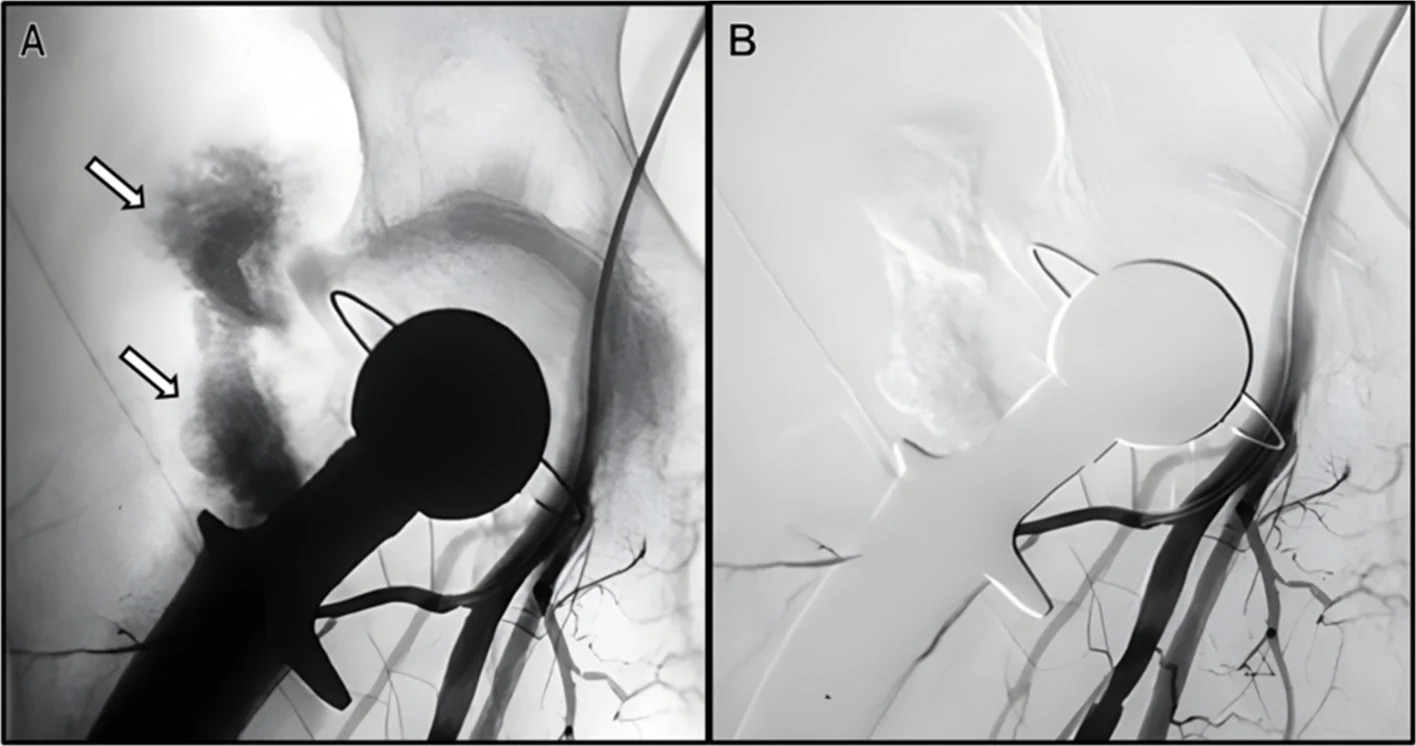
Postoperativeright hip embolization. A 79-year-old woman presenting with an extensive right thigh hematoma and acute blood loss anemia 1 day after total hip arthroplasty. **A **Right hip arteriography. It demonstrates active contrast extravasation (arrows) adjacent to the hip prosthesis. **B **Post-embolization right hip subtraction arteriography. It shows satisfactory occlusion of the right medial circumflex femoral artery achieved using gelatin sponge torpedoes, with a favorable clinical outcome

**Fig. 7:**
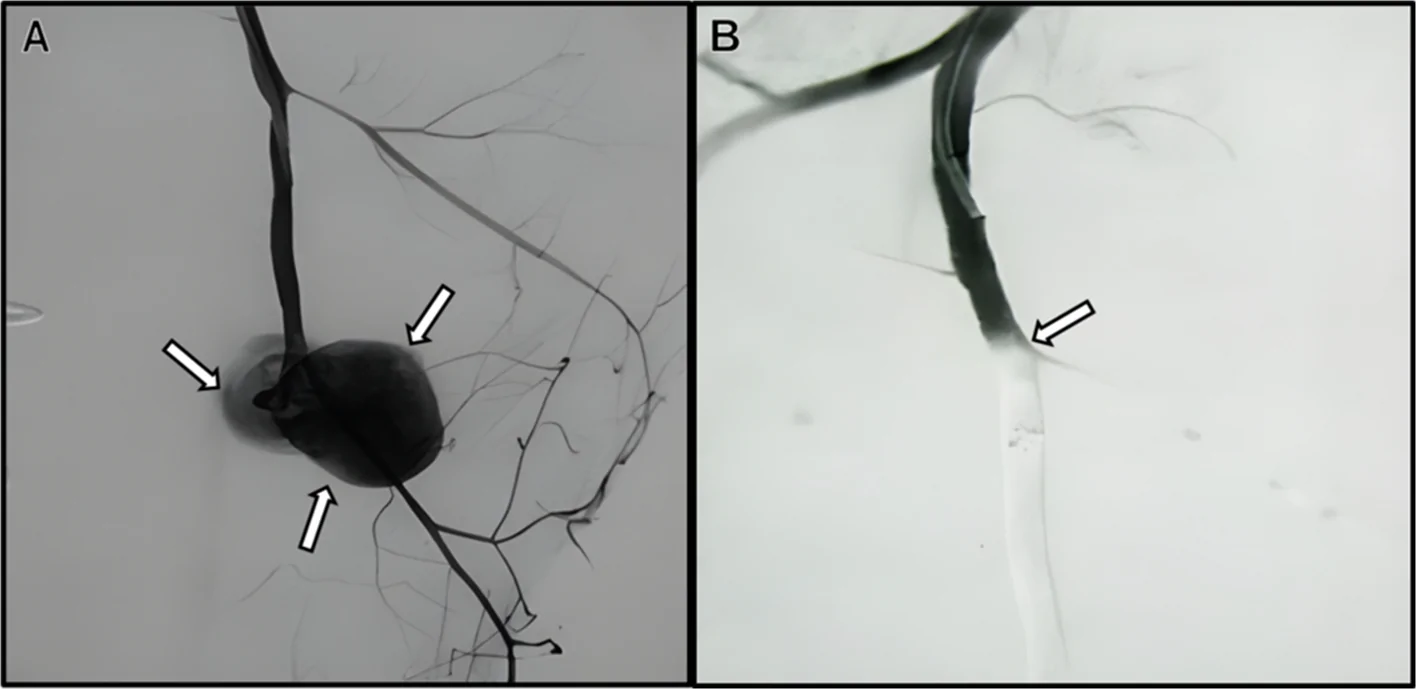
Postoperativeright thigh embolization. A 49-year-old man presenting with a right thigh hematoma and acute blood loss anemia 1 day after right femoral osteotomy. **A **Right femoral subtraction arteriography. It demonstrates contrast stasis consistent with a quadriceps pseudoaneurysm. **B** Postembolizationright femoral subtraction arteriography. It shows satisfactory occlusion achieved using gelatin sponge torpedoes, with a favorable clinical outcome

Postoperative hemorrhage is an uncommon but potentially severe complication of major orthopedic surgery, and TAE can provide minimally invasive hemorrhage control in selected patients. After hip surgery, bleeding or pseudoaneurysm (Figs. 6 and 7) may present with hemodynamic instability, transfusion requirements, increased surgical-drain output, or an expanding thigh hematoma [28].

Transcatheter angiography plays a central role in identifying the bleeding source, and TAE has been shown to be safe and effective in the management of postoperative hemorrhage after hip surgery [28]. Resorbable gelatin sponge (Gelfoam) can be used for proximal occlusion. Endovascular treatment of postoperative pseudoaneurysms may use coils, NBCA, or, in selected complex peripheral vascular injuries, EVOH-based liquid embolics; agent choice depends on anatomy, flow, collateral supply, and operator experience [13, 15].

## Tumor-related embolization

Tumor-related embolization should be interpreted according to therapeutic intent: pre-operative devascularization, symptom palliation, treatment of selected benign hypervascular lesions, or adjunctive use before or during percutaneous ablation or consolidation. The available evidence remains heterogeneous, with variation in tumor type, vascularity, embolization endpoint, timing before surgery, associated treatments, and outcome definitions. Therefore, the magnitude of benefit should be interpreted according to indication and treatment intent rather than generalized across all musculoskeletal tumors.

### Pre-operative embolization of hypervascular bone tumors to reduce bleeding risk

TAE has been used since 1974 for the pre-operative devascularization of hypervascular bone tumors [29]. In particular, TAE of hypervascular spinal bone tumors reduces estimated intraoperative blood loss [3]. Indications for TAE should be discussed in a multidisciplinary setting, taking into account the risk–benefit balance, tumor location, and the arterial territories involved. Reported rates include 93% technical success and 72–100% of post-procedural angiograms demonstrating > 80% tumor devascularization [12, 29].

Hypervascular bone lesions include benign aggressive tumors, primary bone tumors, and metastases, particularly from renal cell carcinoma, thyroid carcinoma, hepatocellular carcinoma, sarcoma, melanoma, and neuroendocrine tumors [12]. Clinical presentation and expected benefit vary according to tumor biology, location, vascularity, and treatment intent.

**Fig. 8:**
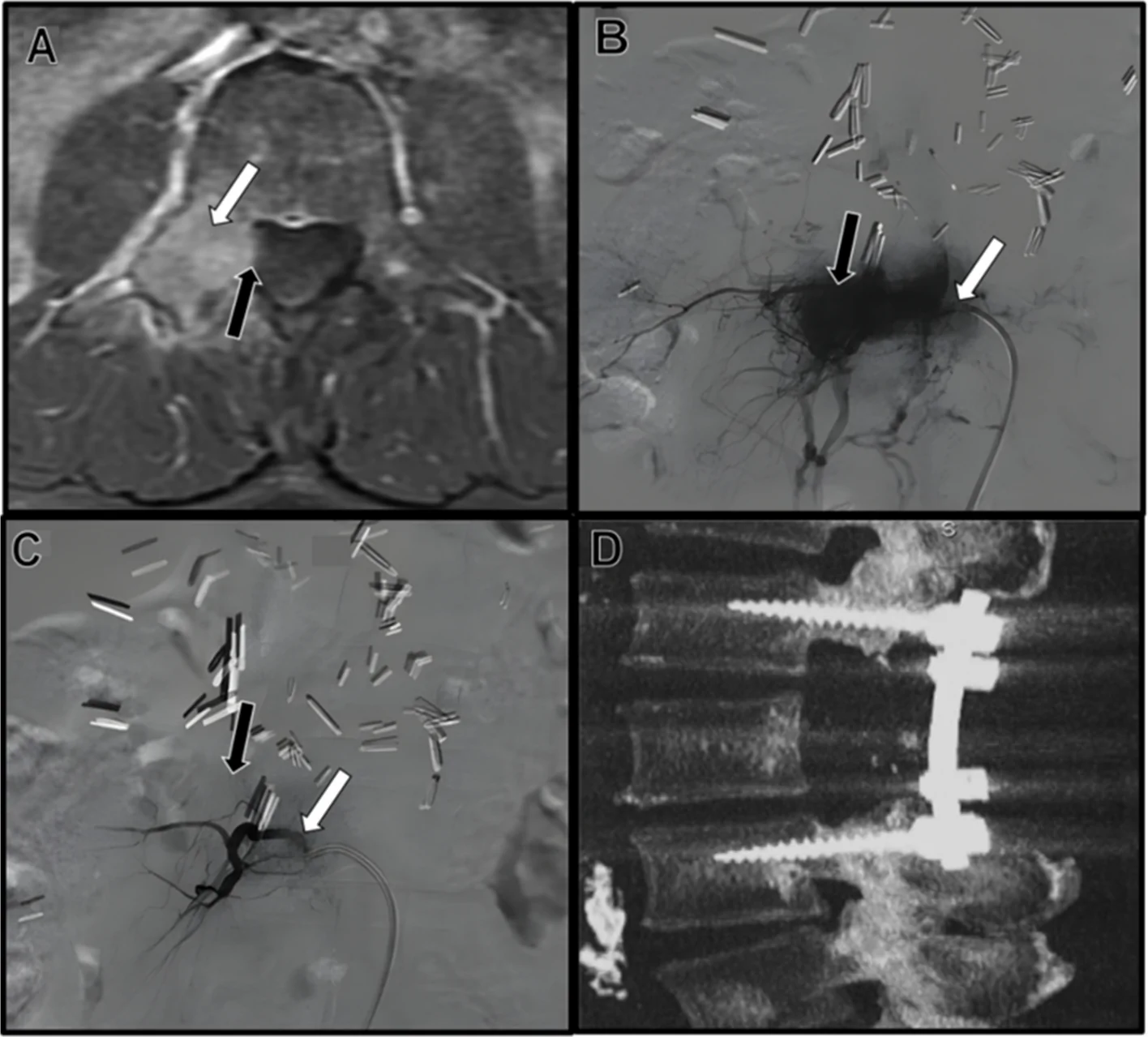
Pre-operative lumbar embolization. **A **Axial contrast-enhanced T1-weighted fat-saturated MRI. It shows a hypervascular bone metastasis in the L3 vertebral body (white arrow) with right-sided epidural extension (black arrow) into the spinal canal, discovered in the context of newly diagnosed renal cell carcinoma. **B **Pre-embolization lumbar angiography. It demonstrates a tumoral blush (black arrow) arising from the right third lumbar artery (white arrow). **C **Post-embolization lumbar angiography. It demonstrates satisfactory pre-operative embolization achieved using calibrated microspheres: the right third lumbar artery (white arrow) remains visible, but the tumoral blush (black arrow) has disappeared. **D** Postoperative lumbar CT (sagittal MIP), after tumor ablation and arthrodesis. It shows that pre-operative embolization enabled minimal blood loss during spine surgery

Pre-operative embolization aims to reduce tumor vascularity, decrease intraoperative blood loss, and facilitate safer resection, usually within 72 h before surgery [3]. An example in vertebral metastases from renal cell carcinoma (RCC) is shown in Fig. 8. Complete evaluation of feeding arteries, collateral pathways, and potential anastomoses to spinal cord, neural, muscular, or cutaneous branches is essential. Embolic-agent selection depends on vascular anatomy, desired depth of devascularization, and safety constraints. Calibrated particles may be used for distal tumor devascularization, whereas coils, plugs, or liquid embolics may be useful for larger feeders or selected high-flow situations. Pain control, patient positioning, and procedural tolerance should also be anticipated, particularly for painful axial lesions.

**Fig. 9:**
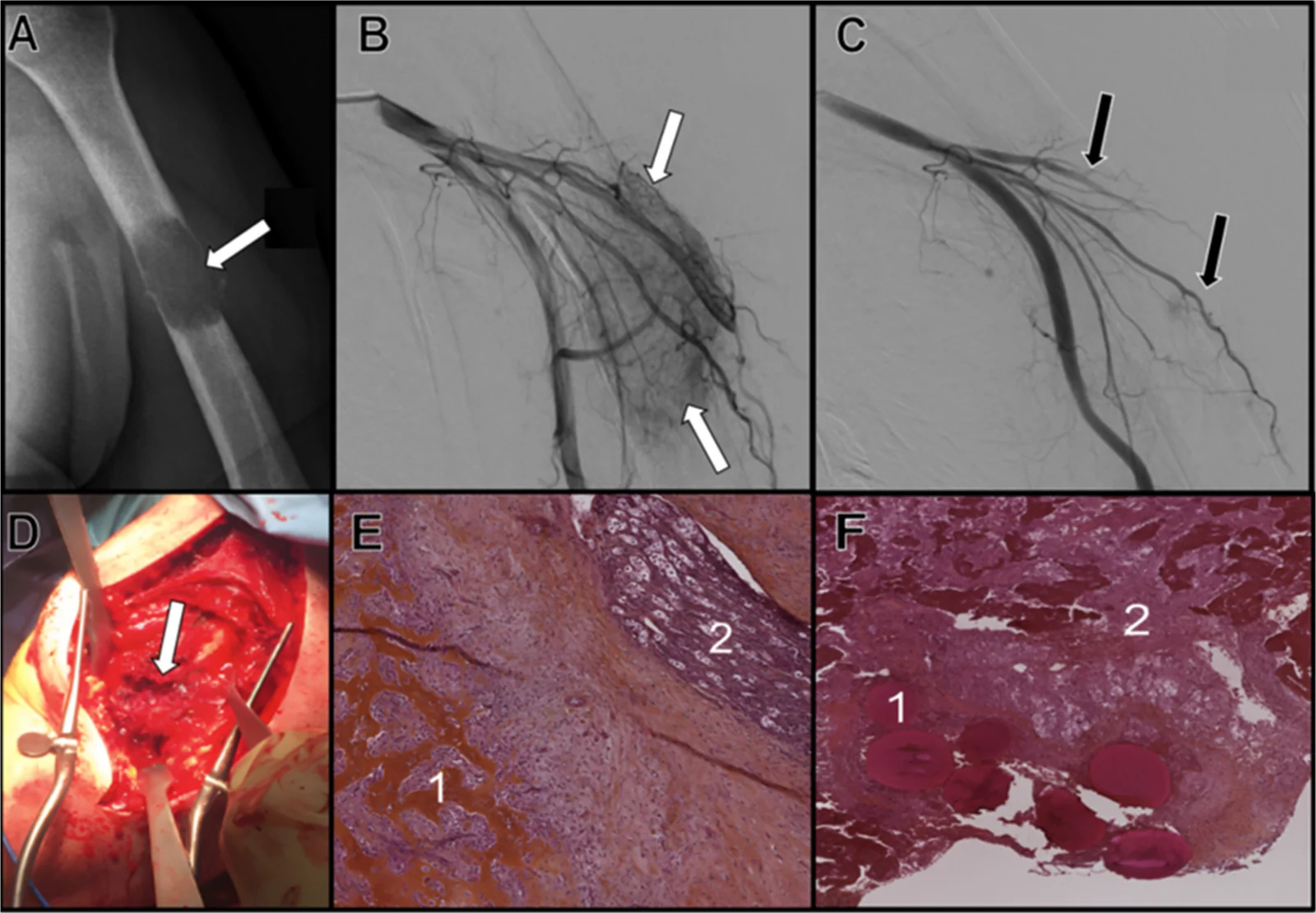
Pre-operative humeral bone embolization. A 65-year-old man presenting with a painful left humeral diaphyseal metastasis from renal cell carcinoma. **A **Radiograph (frontal view). It shows a lytic lesion in the mid-diaphysis of the left humerus (white arrow). **B **Initial arteriography (left upper limb/shoulder). It demonstrates a tumoral blush (white arrows) with multiple feeding arteries. **C** Postembolization arteriography. It demonstrates disappearance of the tumoral blush after embolization with 300–500 µm calibrated microspheres, while the feeding arteries remain visible. **D **Intraoperative photograph. It shows that the bone metastasis (white arrow) can be removed without significant active bleeding. **E **Histology (HES stain, × 20). It shows bone trabeculae (1) and clear cell renal cell carcinoma (2) after embolization and surgery. **F **Histology (HES stain, × 10). It demonstrates 300–500 µm calibrated microspheres within the tumor (1) and within the tumoral infiltration (2) after embolization and surgery

**Fig. 10:**
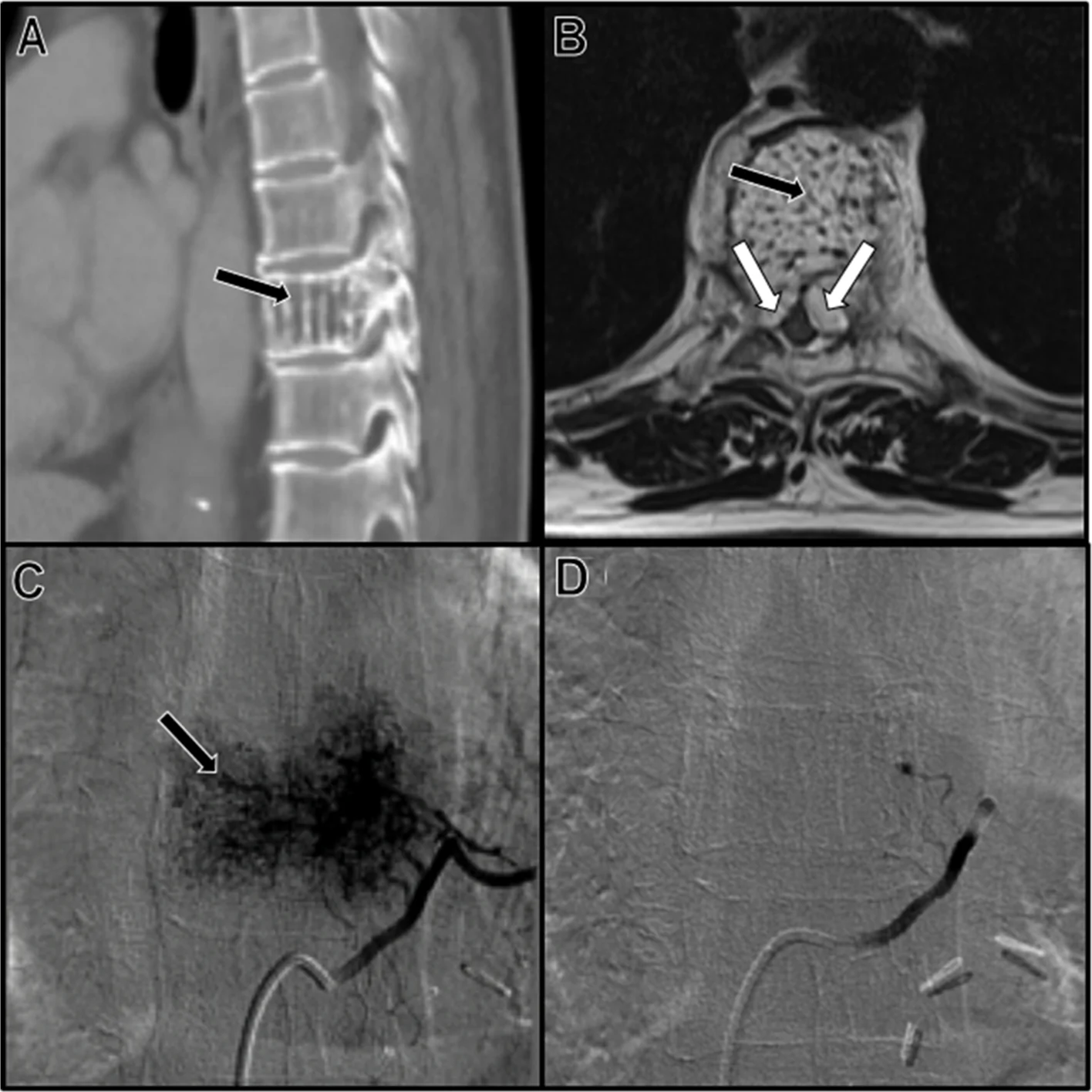
Pre-operative embolization for a thoracic vertebral hemangioma. A 67-year-old man presented with progressive spinal cord compression, a sensory level at the xiphoid process, and pyramidal syndrome of the lower extremities. **A **Pre-embolization CT with sagittal reconstruction. It shows a T8 osseous lesion with a “honeycombing” pattern (black arrow) separated by thickened trabeculae, consistent with a vertebral hemangioma. **B **Pre-embolization axial T2-weighted MRI. It shows abnormal high T2 signal within the vertebral body (black arrow), with intraspinal extension into the spinal canal and the anterior epidural space. **C **Pre-embolization angiography. It shows that superselective catheterization of the left 8th costovertebral artery demonstrates a marked tumoral blush (black arrow), with no radiculomedullary branch identified. **D **Post-embolization angiography. It shows that embolization with 150–250 µm PVA particles achieved satisfactory devascularization involving the entire vertebral lesion

In giant cell tumors confined to the vertebral compartment and in aggressive vertebral hemangiomas associated with spinal cord compression, posterior arch expansion, or pathologic fracture, TAE can also reduce operative blood loss [30, 31]. In 1972, Hekster and colleagues reported that TAE could relieve spinal cord compression caused by vertebral hemangioma [32]. TAE may be effective as sole therapy in selected patients with chronic pain; however, it is not appropriate for extensive lesions causing neurologic symptoms, in which case embolization is performed as preparation for surgery (Figs. 8, 9, and 10 illustrate pre-operative embolization of selected hypervascular bone lesions).

### Embolization as an adjunct to percutaneous tumor therapy

Beyond pre-operative devascularization to reduce intraoperative bleeding, another important role of TAE in oncologic musculoskeletal (MSK) practice is as an adjunct or bridging technique to percutaneous therapies such as radiofrequency ablation (RFA), cryoablation, sclerotherapy, and cementoplasty [30]. By reducing tumor vascularity, TAE can improve the safety and effectiveness of these procedures, particularly in painful hypervascular primary or metastatic bone disease. This strategy is commonly implemented as a staged, two-step approach [3, 11, 30]. The main objective is selective embolization of tumor arterial feeders, thereby lowering the risk of procedural bleeding and reducing the heat-sink effect during RFA. The scope of TAE as part of combination therapy is broad.

In a retrospective single-center study by Sundararajan et al. [33], patients with symptomatic osseous neoplasms underwent embolization followed by cryoablation, with adjunct osteoplasty when mechanical stabilization was required. The authors reported additional improvement in pain scores after sequential treatment; all patients receiving oral analgesics reduced their use, and no complications occurred during embolization, cryoablation, or osteoplasty.

Another reported strategy is the combination of transarterial chemoembolization (TACE) with osteoplasty/cementoplasty. In pelvic bone metastases, Zhang et al. compared percutaneous osteoplasty alone with osteoplasty plus TACE (POPTACE) in 100 patients [5]. Pain and functional outcomes were similar between groups, but early tumor response favored the combined strategy (partial response at 1 month: 74% with POPTACE vs 52% with osteoplasty alone). Finally, Grange et al. [30] proposed a combined approach for aggressive vertebral hemangioma in which staged embolization and percutaneous radiopaque gelified ethanol sclerotherapy, followed by vertebroplasty, was technically feasible and generally effective for pain relief. These studies suggest that TAE can serve as a practical enabler of multimodality percutaneous MSK tumor care—particularly for hypervascular tumors and complex pelvic/axial disease—while underscoring the need for meticulous technique in sensitive territories such as spinal embolization. TAE may therefore facilitate broader multimodal strategies.

### Palliative embolization for pain

**Fig. 11:**
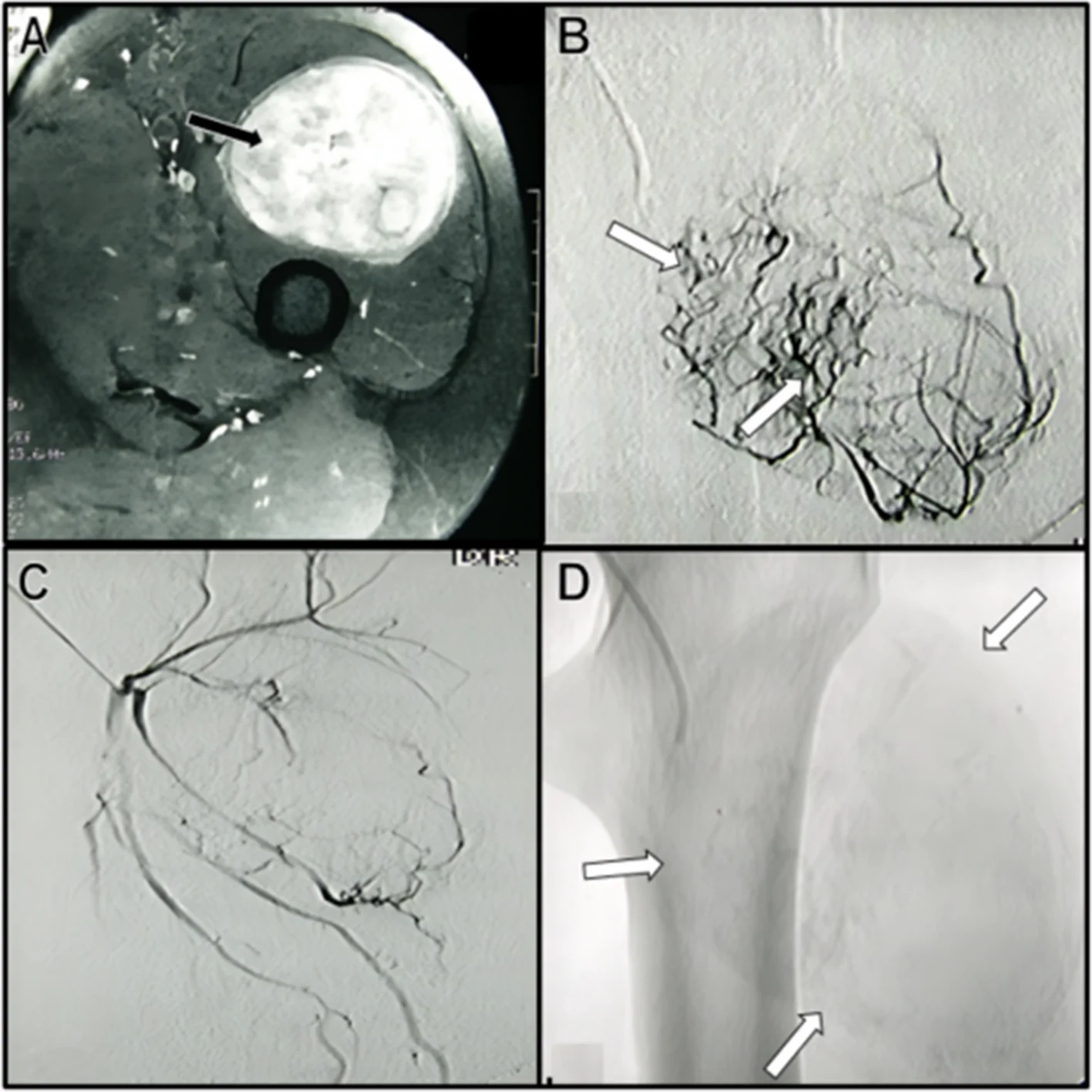
Palliative embolization of a left thigh soft-tissue sarcoma in a 33-year-old man. **A **Axial T1-weighted fat-suppressed MRI after gadolinium administration. It shows a hypervascular tumor of the left quadriceps presenting as a large enhancing mass. **B **Pre-embolization angiography. It demonstrates numerous abnormal tortuous vessels associated with a marked tumoral blush (white arrows). **C** Postembolization angiographyafter superselective embolization. It shows that injection of calibrated microspheres (500–700 µm) resulted in satisfactory devascularization, with preservation of the main arterial trunks and complete disappearance of the tumoral blush and dysplastic arterioles. **D** Postembolization angiography with osseous landmarks. It shows retention of iodinated contrast material within the tumor (white arrows)

In nonsurgical candidates, TAE may also be considered for symptomatic patients (Fig. 11). The goals of TAE are to control intractable pain refractory to major analgesics, improve quality of life, and facilitate combined approaches with thermoablation and consolidative procedures (e.g., screw fixation and cementoplasty). Embolization of primary or secondary bone tumors should be as selective as possible, typically using nonresorbable particles such as calibrated microspheres. Smaller particles may be more effective but increase the risk of ischemia and necrosis in adjacent normal tissues.

In a retrospective series of 164 patients with spinal metastases, Facchini et al. reported a reduction of at least 50% in pain scores and analgesic requirements in 159 patients (97%), with a mean duration of pain relief of 9.2 months [34]. Because this evidence derives from a single retrospective cohort, it should not be generalized to all musculoskeletal tumors. Transarterial chemoembolization has also been used for osteosarcoma, with histologic tumor devascularization reported in 70–94% of specimens [35]. Reported adverse events include post-embolization syndrome, skin or soft-tissue necrosis, neurologic complications, and arterial injury; their frequency varies by tumor location, vascular anatomy, embolic agent, and treatment intent [3, 12].

## Expanding non-oncologic indications

### Embolization in recurrent hemarthrosis or chronic joint bleeding

Hemarthrosis has multiple etiologies, including trauma, anticoagulant therapy, bleeding disorders (e.g., hemophilia [4]), inflammatory or degenerative arthritis, and iatrogenic causes. Recurrent spontaneous hemarthrosis after joint replacement is rare, with a reported incidence of 0.17–1.6% [36]. It is typically related to hypervascular hypertrophic synovitis and may resolve spontaneously. Recurrence after conservative management is low; conservative measures include joint immobilization, ice application, analgesics/anti-inflammatory drugs, selective aspiration, and adjustment or temporary cessation of anticoagulation. In the event of recurrence, arthroscopic synovectomy or open surgical management (e.g., vessel ligation/clipping or thermoablation) can be effective. However, these procedures may be time-consuming, carry an infectious risk, and may be associated with perioperative bleeding if the culprit arterial pedicles cannot be identified. Radionuclide synoviorthesis using intra-articular yttrium-90 may be considered, although long-term risks remain uncertain.

**Fig. 12:**
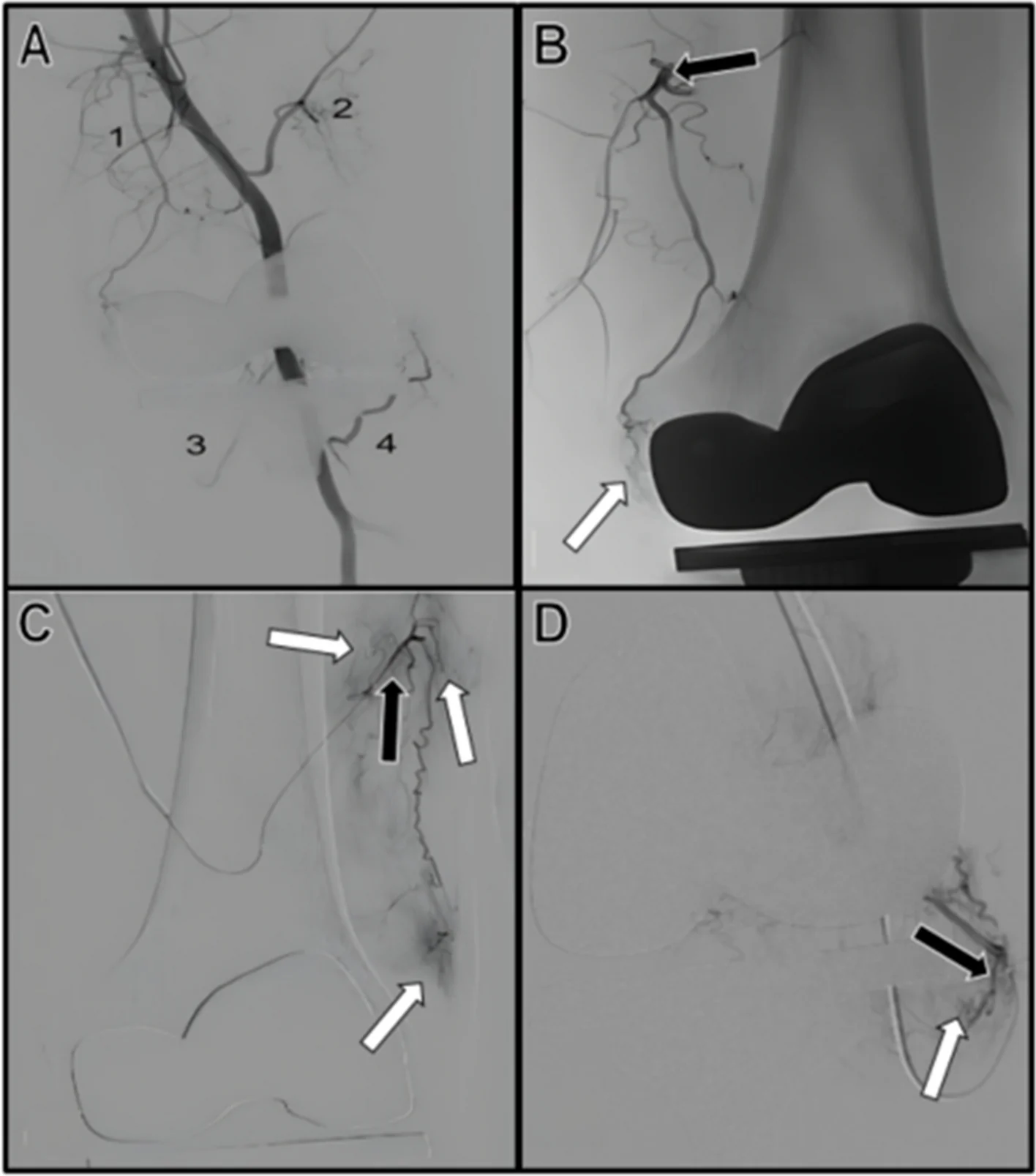
Embolization for recurrent hemarthrosis after left total knee arthroplasty in a 55-year-old man. **A** Digital subtraction angiography (DSA) of the left knee. It depicts the descending genicular artery (1), the superior lateral genicular artery (2), the inferior medial genicular artery (3), and the inferior lateral genicular artery (4). **B–D** Superselective catheterization. It shows the descending genicular artery (**B**), superior lateral genicular artery (**C**), and inferior lateral genicular artery (**D**) (black arrows). DSA demonstrates an abnormal hypervascular blush with associated synovial hyperemia (white arrows)

**Fig. 13:**
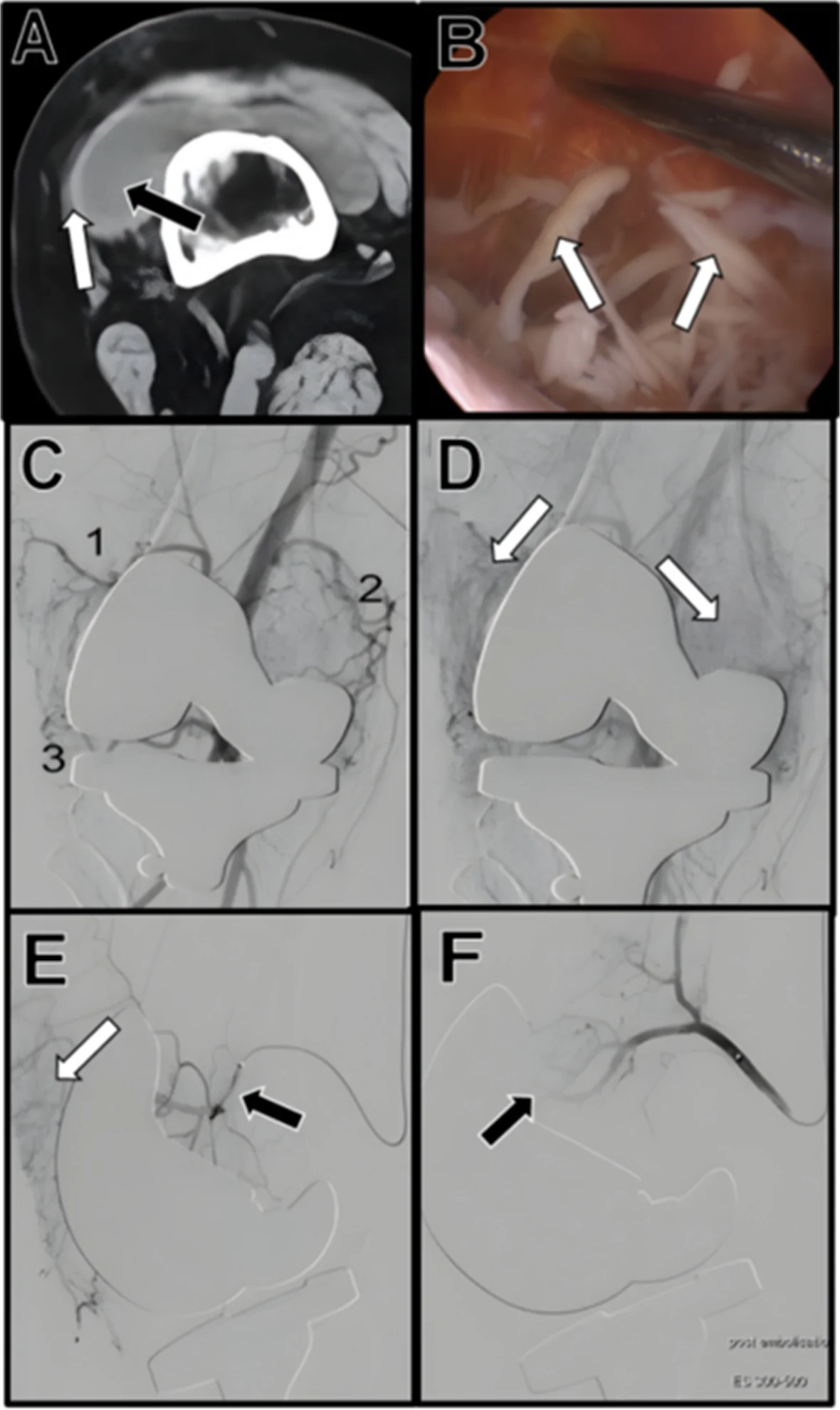
Genicular artery embolization in a 58-year-old woman with rheumatoid arthritis diagnosed 30 years earlier. A right total knee arthroplasty was performed for refractory pain. She subsequently developed recurrent hemarthrosis (synovial biopsy showing rheumatoid inflammatory changes) despite radiosynoviorthesis and synovectomy. **A **Axial CT images. They show intra-articular fluid (black arrow) and capsular thickening (white arrow) in the right knee. **B** Arthroscopic view. It demonstrates hypertrophic synovial fringes/villi (white arrow). **C **Anteroposterior (AP) angiography before embolization (early phase). It depicts the (1) superior lateral, (2) superior medial, and (3) inferior lateral genicular arteries. **D **Frontalangiography before embolization (delayed phase). It demonstrates pathological synovial hyperemia of the right knee. **E **Lateral angiography before embolization after superselective catheterization of the superior lateral genicular artery (black arrow). It shows an inflammatory blush (white arrow). **F **Lateralangiography after embolization. After using calibrated microspheres (from 300 to 500 μm), stasis of the targeted vessels (black arrow) was obtained. Clinical improvement was rapidly observed after embolization

Genicular artery embolization (GAE) is a safe and effective treatment for spontaneous hemarthrosis of the knee after arthroplasty [36]. A detailed understanding of genicular arterial anatomy is essential for safe and successful procedures [8] (Figs. 1, 12, and 13).

In a recent review of non-hemophilic recurrent hemarthrosis, technical success was achieved in 99% of patients; the postembolization recurrent bleeding rate was 10%, with a mean time to recurrence of 8.1 months [36]. Major complications occurred in 2% of cases (mainly joint infections), and minor complications occurred in 6%, including transient skin ischemia, ulceration, or pain. Notably, all transient skin complications occurred after use of spherical embolic particles < 300 µm or when n-butyl cyanoacrylate (NBCA) glue was used; therefore, spherical particles > 300 µm are recommended. However, proximal coil placement should be avoided because distal revascularization may occur and subsequent arterial access can be compromised in the event of recurrence. In patients with hemophilia, TAE can significantly reduce the number of bleeding episodes over the following year [4].

### Stand-alone pain-directed embolization for degenerative joint disease and chronic inflammatory tendinopathies

In contrast to hemostatic or tumor-related indications, pain-directed embolization for osteoarthritis and chronic inflammatory tendinopathies is generally performed as a stand-alone minimally invasive therapeutic procedure. The therapeutic rationale is to reduce pathological neovascularity and associated neo-innervation rather than to serve as an adjunct to surgery, ablation, or another percutaneous intervention. This distinguishes TAE from traditional multimodal management, which typically combines analgesics, physiotherapy, activity modification, intra-articular or peritendinous injections, and, in selected advanced cases, surgery. In this setting, embolization is proposed for carefully selected refractory patients as a targeted vascular intervention rather than as a component of an operative pathway.

TAE for chronic degenerative and inflammatory tendinopathies is based on the concept that these conditions are driven, at least in part, by pathologic neovascularization and persistent synovial or peritendinous hyperemia within inflamed tissues [8]. These newly formed, fragile vessels may sustain inflammatory signaling and serve as conduits for nociceptive nerve ingrowth, thereby amplifying pain and promoting chronicity [8]. By selectively catheterizing and embolizing these abnormal neovessels, TAE aims to reduce regional hypervascularity, decrease the delivery of inflammatory mediators, and promote regression of associated perivascular nerves. This targeted vascular approach is intended to alleviate pain and improve function while preserving normal tissue perfusion. On angiography, these abnormal vascular networks may appear as a focal hyperemic “blush” (sometimes described as a tumor-like blush). Building on this rationale, we discuss the clinical applications investigated to date, including GAE, embolization for adhesive capsulitis, and targeted tendinous embolization.

#### Genicular artery embolization (GAE) in knee osteoarthritis (OA) and persistent pain or hemarthrosis after arthroplasty

GAE is a more recent application of TAE, based on the premise that embolization of abnormal periarticular neovessels may lead to regression of accompanying nociceptive nerve fibers [8].

Typical inclusion criteria include symptomatic knee osteoarthritis with persistent pain despite optimized conservative treatment, concordance between symptoms, clinical examination, and imaging findings, and angiographic evidence of abnormal periarticular hypervascularity corresponding to the painful compartment. Most studies have focused on mild-to-moderate radiographic osteoarthritis rather than end-stage disease. Potential exclusion criteria include advanced end-stage osteoarthritis requiring arthroplasty, severe malalignment or mechanical instability as the dominant pain driver, active infection, inflammatory arthropathy not controlled medically, severe peripheral arterial disease, uncorrectable coagulopathy, renal dysfunction limiting contrast administration, pregnancy, and inability to complete standardized clinical follow-up.

**Fig. 14:**
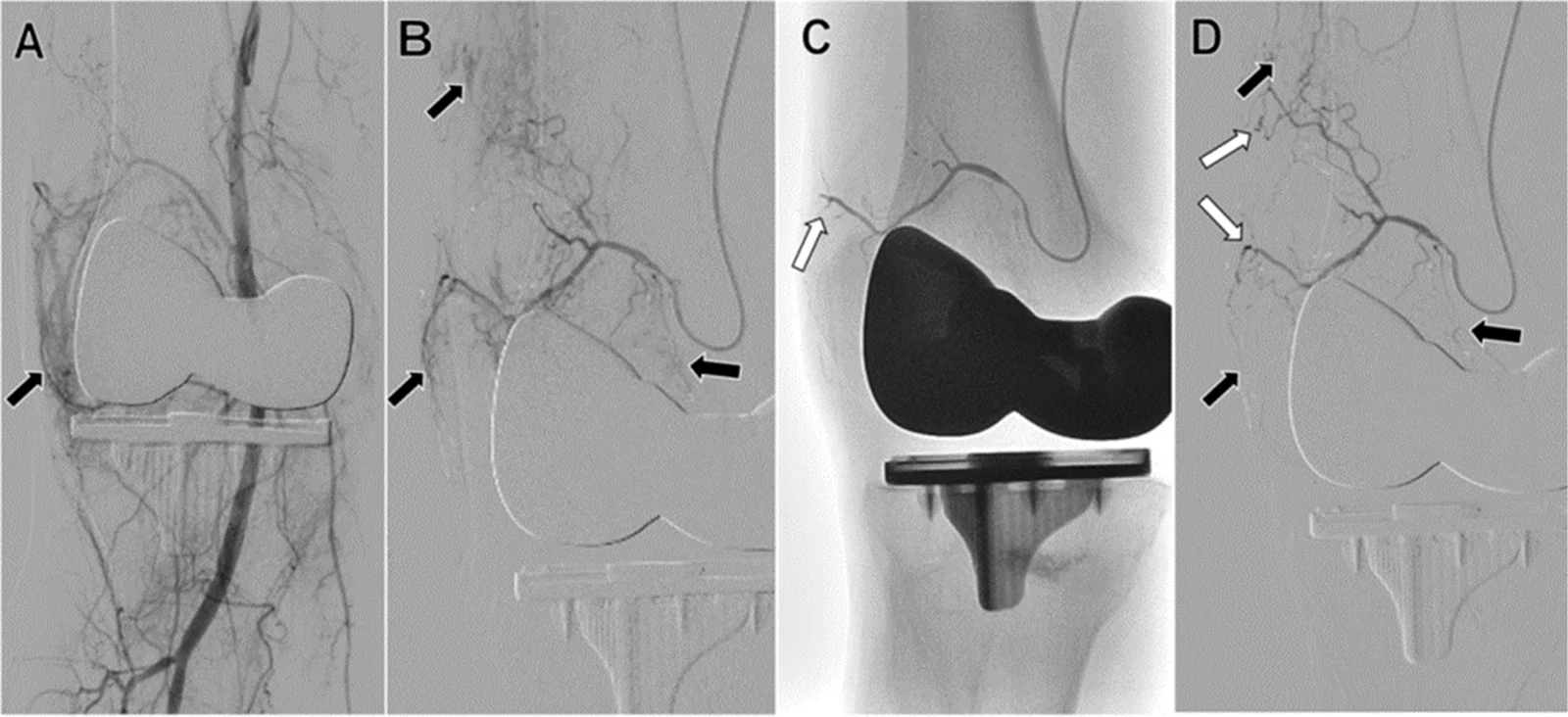
Genicular artery embolization in a 54-year-old man with persistent pain after right total knee arthroplasty. **A **Anteroposterior (AP) angiography before embolization (delayedphase). It demonstrates pathological synovial hyperemia of the right knee with an inflammatory blush (black arrow). **B **Frontal angiography before embolization after superselective catheterization of the superior lateral genicular artery (SLGA) (delayedphase). It confirms the pathological synovial hyperemia, with several areas of inflammatory blush supplied by the SLGA (black arrows). **C** Frontalangiography during embolization. A mixture of Lipiodol and iodinated contrast medium is injected under fluoroscopic guidance into the SLGA. **D** Frontalangiography after embolization (delayed phase). It shows stasis/occlusion of the targeted vessels (white arrows) and disappearance of the blush. Clinical improvement was rapidly observed after embolization and was maintained at 1-year follow-up

Technical success should be defined as superselective catheterization of the target genicular or periarticular branches and embolization of angiographically abnormal hypervascularity without major non-target embolization. Clinical success should be defined using prespecified patient-reported outcomes, such as improvement in pain and function scores, reduction in analgesic use, and patient global assessment at standardized follow-up intervals. Patient evaluation should include baseline pain assessment, functional scoring, radiographic grading, assessment of prior conservative treatments, documentation of analgesic use, and follow-up at standardized intervals, commonly 1, 3, 6, and 12 months. Longer-term follow-up is needed to determine durability and the potential need for repeat embolization or subsequent arthroplasty. Access is typically radial or femoral, followed by selective catheterization of the genicular arteries. Targeted embolization is performed at sites of abnormal hyperemic blush on angiography (Fig. 14). Embolic agents reported in the literature include 100–300 µm microspheres, embozene particles, imipenem–cilastatin (IMP/CIS) [7] slurry, and lipiodol-based [19] preparations, delivered into genicular branches to reduce synovial hypervascularity while sparing normal periarticular flow. The LipioJoint-1 trial, a prospective multicenter single-arm study, reported meaningful pain relief and functional improvement with a favorable safety profile, supporting Lipiodol-based GAE as a minimally invasive option for symptomatic knee OA [19]. Technical success is consistently high across published series [37].

Several prospective cohorts and single-arm trials have reported high technical success and improvements in pain and function after GAE, sometimes lasting 12–24 months [19, 38, 39]. These findings are also summarized in recent technical and systematic reviews [8, 37]. However, sham-controlled evidence is inconsistent. Van Zadelhoff et al. found no significant benefit over sham at 4 months [40]; in the subsequent 12-month report, both groups had sustained pain improvement, with no significant between-group differences in pain, synovitis, or other clinical outcomes, leading the authors to conclude that the findings did not support routine clinical implementation of GAE [41].

In contrast, the 2026 Society of Interventional Radiology position statement supports GAE as a viable joint-preserving option for appropriately selected patients with symptomatic knee osteoarthritis who have failed conservative treatment and are not candidates for, or wish to delay, arthroplasty, while also calling for larger randomized trials [42]. Taken together, these data support a cautious, individualized interpretation rather than routine use across all patients.

In a prospective single-arm study, Taslakian et al. [39] evaluated GAE in symptomatic knee OA and reported sustained clinical improvement along with associated biologic changes. At 12-month follow-up, significant reductions in circulating vascular endothelial growth factor (VEGF) and interleukin-1 receptor antagonist (IL-1Ra) were observed, supporting a potential role of GAE in modulating neoangiogenesis and inflammatory pathways implicated in OA-related pain. Jin-Woo Choi et al. [43] assessed the value of pre-procedural MRI in patients undergoing GAE for knee OA pain. MRI findings—particularly synovitis, bone marrow lesions, and contrast-enhancing synovial hypertrophy—were associated with better clinical response after GAE, whereas advanced structural degeneration appeared to derive less benefit. These findings support MRI as a tool for patient selection and prognostication, consistent with the concept that GAE may be most effective in knees with active inflammatory and hypervascular features rather than end-stage degenerative disease.

GAE has also been investigated for chronic pain after total knee arthroplasty. Early clinical studies suggest technical feasibility and potential improvement in selected refractory patients, but the evidence remains limited and should be interpreted cautiously [44, 45].

Beyond the knee, pain-directed embolization has also been considered for other joints, including the hip, temporomandibular joint, interphalangeal joints, thumb base, and facet joints. These applications remain early and hypothesis-generating. They may broaden the concept of joint embolization, but each vascular territory has specific safety constraints and requires anatomical validation, standardized imaging criteria, and prospective clinical assessment before routine clinical use.

#### Epicondylitis

**Fig. 15:**
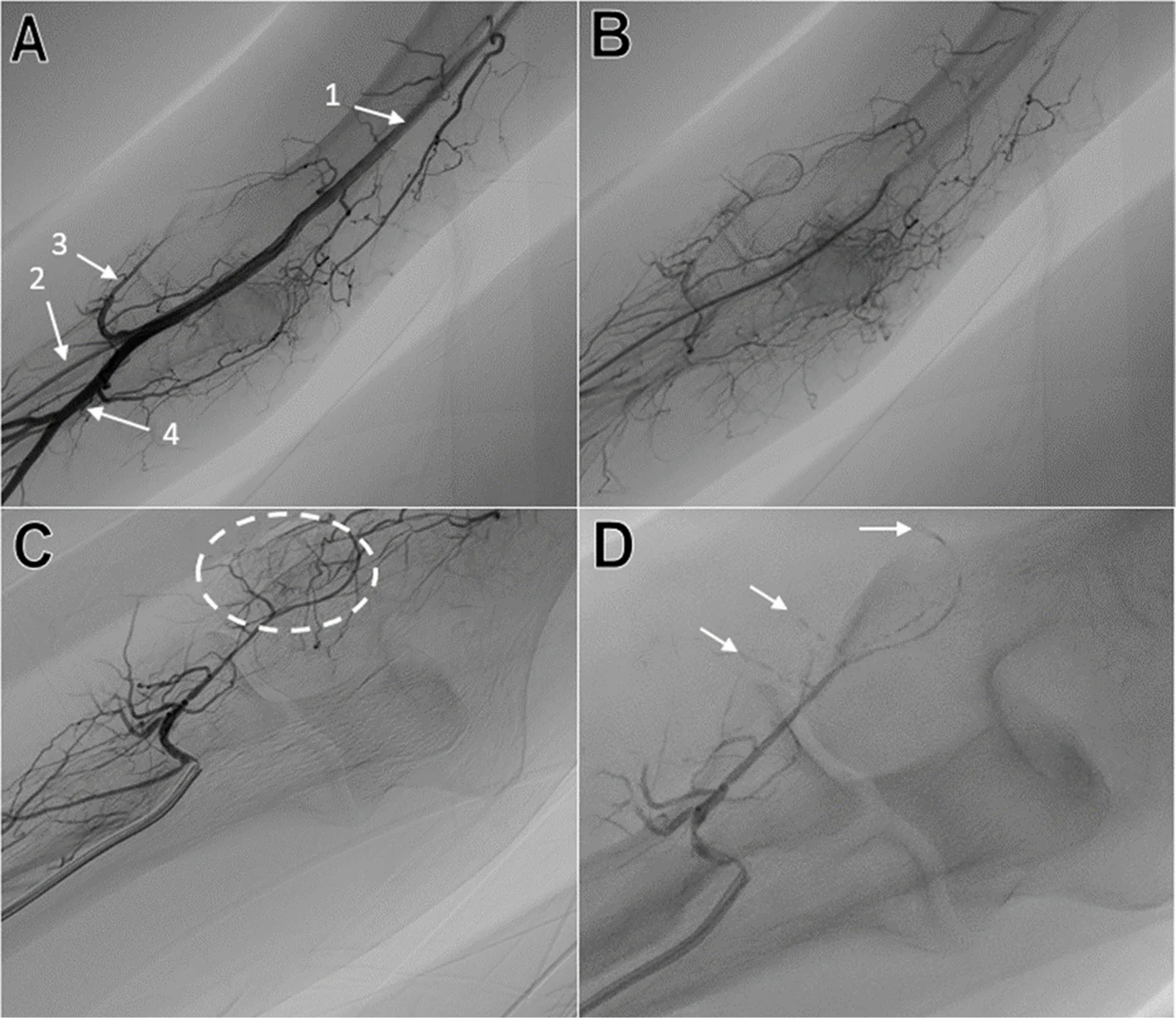
Embolization of the right elbow for refractory lateral epicondylitis in a 40-year-old woman. The patient presented with persistent pain (VAS 7/10) despite first-line treatments, resulting in professional impairment. Right elbow angiography was performed via radial access. **A** Angiography, early arterial phase. 1 = brachial artery; 2 = radial artery; 3 = recurrent radial artery; 4 = ulnar artery. **B** Angiography, delayed phase. It shows no obvious inflammatory blush. **C** Angiography of the recurrent radial artery, after selective catheterization. An inflammatory blush is demonstrated (dashed circle), consistent with pathologic hypervascularization in the setting of chronic epicondylitis. **D** Angiography of the recurrent radial artery after embolization using a Lipiodol/iodinated contrast mixture. Stagnation of the mixture is observed within the recurrent radial artery and its distal branches (white arrows), indicating occlusion of the pathological microvasculature. At 1-month follow-up, the VAS score decreased to 3/10

Lateral and medial epicondylitis are overuse tendinopathies related to repetitive forearm strain and are a common cause of elbow pain. Both entities are primarily degenerative and are characterized histologically by fibroblastic degeneration and pathologic neovascularization with accompanying perivascular nerve ingrowth [46, 47]. Standard management is similar to that of other chronic tendinopathies and may include physical therapy, nonsteroidal anti-inflammatory drugs, corticosteroid injections, and, in selected refractory cases, surgical treatment. More recently, TAE has emerged as an alternative approach aimed at embolizing these abnormal neovessels [48] (Fig. 15). Target vessels are selected according to the symptomatic compartment and angiographic hyperemic blush, with careful attention to muscular, cutaneous, and neural branches. Embolic agents reported include PVA particles, gelatin sponge, and imipenem–cilastatin slurry.

In the prospective 24-patient pilot study by Iwamoto et al., mean maximum-pain VAS decreased from 77± 15 mm at baseline to 16 ± 13 mm at 6 months, 9 ± 13 mm at 12 months, and 11 ± 14 mm at 24 months [48]. Postprocedural MRI findings also showed improvement in tendinosis without evidence of muscle loss or cartilage or bone necrosis [48]. These findings are encouraging but derive from a small uncontrolled cohort and require confirmation in comparative trials. No serious adverse events have been reported in the available small series; transient postprocedural pain, generally resolving within 48–72 h, has been described [48, 49]. Overall, early uncontrolled studies suggest that TAE may provide pain relief in selected refractory patients, but comparative effectiveness and recurrence rates relative to conservative treatment remain uncertain. Data for medial epicondylitis remain limited. Lee et al. evaluated TAE in 10 patients with refractory medial epicondylitis and reported significant pain reduction without major adverse events [50].

#### Plantar fasciitis and Achilles tendinopathy

Plantar fasciitis and Achilles tendinopathy have also been investigated using embolization, based on the rationale of pathological neovascularity and nociceptive nerve ingrowth [51, 52]. However, the evidence remains limited and heterogeneous. For plantar fasciitis, causal interpretation is particularly challenging because spontaneous improvement may occur within several months in many patients. In the largest reported plantar fasciitis cohort, Sasaki et al. [53] described 66 cases with up to 4 years of follow-up, but most procedures were ultrasound-guided and patients underwent a mean of 2.3 reinterventions. Future studies should focus on clearly refractory cases, standardized imaging criteria, and comparative outcomes.

#### Adhesive capsulitis

Adhesive capsulitis is an idiopathic inflammatory condition classically described as progressing through three phases: freezing, frozen, and recovery. It is thought to be triggered by soft-tissue injury, initiating an inflammatory cytokine cascade that promotes neovascularization and accompanying nociceptive nerve ingrowth [54]. Traditional management includes oral analgesics, physical therapy, intra-articular injections, and, in selected cases, arthroscopic capsular release [55]. More recently, TAE has been investigated as an alternative treatment [56]. The goal of TAE is to embolize hyperemic areas harboring abnormal neovessels, thereby attenuating the inflammatory response and targeting associated perivascular nerves. Understanding the arterial supply of the shoulder is essential for safe TAE. The main contributors include the suprascapular, anterior and posterior circumflex humeral, thoracoacromial, and subscapular arteries. In adhesive capsulitis, abnormal neovessels have been described as newly developed periarticular microvessels arising in addition to the normal capsular supply [57]. Diagnosis is primarily clinical, supported by imaging [58]. Selected patients may undergo angiography to identify abnormal hyperemic “blush”-type enhancement prior to targeted embolization [59, 60].

The most commonly reported embolic agents for adhesive capsulitis are resorbable materials and imipenem–cilastatin (IMP/CIS) slurry [56]. A recent multicenter study [61] found that TAE for shoulder musculoskeletal pain using an ethiodized oil-based emulsion appeared feasible and safe, supporting further evaluation in larger prospective cohorts. Nevertheless, embolization for adhesive capsulitis remains exploratory. Early studies suggest potential improvements in pain and range of motion, but the natural history of the disease, small sample sizes, and limited comparative data prevent firm conclusions regarding treatment effect.

## Discussion

The main finding of this narrative review is that musculoskeletal transarterial embolization (TAE) should not be considered a single homogeneous field. Its indications differ markedly in clinical maturity, evidence quality, therapeutic intent, and expected durability. Hemostatic and selected tumor-related applications are established within multidisciplinary pathways, whereas stand-alone pain-directed procedures for osteoarthritis and tendinopathies remain promising but less mature and require stricter patient selection and standardized outcome assessment (Table 2).

**Table 2:** Evidence maturity and clinical status of musculoskeletal transarterial embolization indications. The categories “established,” “intermediate,” “emerging,” “exploratory,” and “hypothesis-generating” were developed by the authors for this narrative review, based on therapeutic intent, study design, sample size, consistency of outcomes, duration of follow-up, comparative evidence, safety data, and current clinical use. They do not represent a formally validated evidence-grading system or a guideline/consensus classification

Indication	Evidence maturity	Main evidence base	Primary outcomes	Durability	Adverse events/limitations	Current status
Trauma and post-operative hemostasis	Established	Mostly retrospective series, trauma registries, technical reports; embedded in multidisciplinary trauma pathways	Rapid hemorrhage control; reduced transfusion/surgical need in selected patients	Acute follow-up; outcomes often confounded by injury severity	Non-target embolization, access complications, tissue ischemia; generally acceptable when superselective	Routine clinical use in appropriate settings
Tumor-related embolization	Established to intermediate, depending on intent	Heterogeneous series; strongest for selected hypervascular tumors and preoperative devascularization	Reduced vascularity/bleeding; palliation or adjunct to other procedures	Variable; depends on tumor type, surgery timing, and combined therapy	Post-embolization pain, non-target ischemia, incomplete devascularization	Use in multidisciplinary tumor boards; individualized indications
Recurrent hemarthrosis	Intermediate	Small to moderate clinical series; mostly uncontrolled	Reduction of recurrent bleeding episodes and improved symptoms	Variable; recurrence may require repeat treatment	Non-target cutaneous or periarticular ischemia is uncommon but relevant	Clinically used in selected refractory cases
GAE for knee osteoarthritis	Emerging; professional-society support but conflicting sham-controlled evidence	Prospective cohorts, meta-analyses, sham-controlled randomized trials, and a 2026 SIR position statement	Improvement in uncontrolled studies; no sustained superiority over sham in some randomized trials	Reported to 12–24 months in cohorts; 12-month sham-controlled data show similar improvement in both groups	Skin discoloration, transient pain, non-target embolization; agent-specific considerations	Potential option for carefully selected patients; routine use remains debated
Adhesive capsulitis	Exploratory/emerging	Small or early clinical cohorts	Reported pain and range-of-motion improvement	Natural history and spontaneous improvement complicate causality	Transient pain or inflammatory symptoms; non-target risk	Requires prospective comparative validation
Epicondylitis	Exploratory/emerging	Small cohorts; limited comparative data	Reported symptom improvement in refractory cases	Short/variable follow-up	Non-target embolization to cutaneous/muscular/neural branches	Potential stand-alone option; investigational
Plantar fasciitis and Achilles tendinopathy	Exploratory	Small cohorts/case series; limited randomized evidence	Possible pain improvement in refractory cases	Spontaneous improvement over months may confound interpretation	Ischemic and non-target risks require careful technique	Research/selected cases only
Extra-knee joint OA or pain: hip, TMJ, interphalangeal, thumb base, facet joints	Hypothesis-generating	Very limited early reports or conceptual extrapolation	Not yet standardized	Durability unknown	Joint-specific vascular safety not fully defined	Needs anatomical, safety, and prospective clinical validation

*Abbreviations*: *ABC* aneurysmal bone cyst, *EVOH* ethylene–vinyl alcohol copolymer, *GAE* genicular artery embolization, *NBCA* n-butyl cyanoacrylate, *OA* osteoarthritis, *PVA* polyvinyl alcohol, *TAE* transarterial embolization, *TMJ* temporomandibular joint

In acute hemorrhage, the clinical value of TAE depends on rapid integration with resuscitation, imaging, correction of coagulopathy, surgery or packing when required, and post-operative monitoring. Relevant outcomes therefore extend beyond angiographic occlusion to hemodynamic stabilization, transfusion requirements, repeat intervention, ischemic complications, and mortality. For tumor-related indications, benefit is similarly context-dependent and should be interpreted according to tumor vascularity, treatment intent, embolization endpoint, and integration with surgery, ablation, cementoplasty, radiotherapy, or systemic therapy.

Pain-directed embolization differs because it is used as the principal therapeutic intervention. Although observational studies report improvements in selected patients, chronic pain is influenced by structural disease, natural history, concomitant therapy, central sensitization, and placebo effects. For GAE, uncontrolled cohort data must be balanced against conflicting sham-controlled trials, including 12-month evidence showing no superiority over sham. The 2026 Society of Interventional Radiology position statement nevertheless considers GAE a viable option for appropriately selected patients after failed conservative therapy, while calling for larger randomized trials. Other pain-directed indications remain supported mainly by small uncontrolled series.

Across emerging indications, the main limitations are small samples, heterogeneous selection criteria and embolic agents, inconsistent outcome definitions, and limited long-term comparative data. Future studies should use indication-specific technical, clinical, and safety endpoints, with particular emphasis on patient-centered outcomes, durability, analgesic use, and subsequent surgery.

Future development should remain indication-specific: pragmatic pathway-based evaluation for established hemostatic and selected oncologic uses, and rigorous comparative assessment for pain-directed procedures.

This review has limitations. It was designed as a narrative review and not as a systematic review or meta-analysis. Although a structured literature search was performed, no formal risk-of-bias assessment or quantitative synthesis was undertaken. The level of evidence was therefore summarized qualitatively, with emphasis on clinical maturity, study design, follow-up, comparative data, and current clinical status.

## Conclusion

Transarterial embolization has an expanding but indication-dependent role in musculoskeletal care. Hemostatic and selected tumor-related applications are established components of multidisciplinary management. For knee osteoarthritis, uncontrolled prospective cohorts have reported clinically meaningful improvements, but randomized sham-controlled evidence remains inconsistent, including 12-month data showing no sustained superiority over sham. Other stand-alone pain-directed applications, including tendinopathies and adhesive capsulitis, remain exploratory. Future studies should prioritize standardized patient selection, procedural reporting, comparative outcomes, safety assessment, and long-term durability.

## Data Availability

Data sharing is not applicable to this article as no datasets were generated or analyzed during the preparation of this review.

## Abbreviations

DGA: Descending genicular artery
GAE: Genicular artery embolization
IMP/CIS: Imipenem/cilastatin
LIGA: Lateral inferior genicular artery
LSGA: Lateral superior genicular artery
MCID: Minimal clinically important difference
MIGA: Medial inferior genicular artery
MSGA: Medial superior genicular artery
MSK: Musculoskeletal
NBCA: N-butyl cyanoacrylate
OA: Osteoarthritis
PEG: Polyethylene glycol
PRP: Platelet-rich plasma
PVA: Polyvinyl alcohol
RCC: Renal cell carcinoma
RCT: Randomized controlled trial
ROM: Range of motion
TAE: Transarterial embolization
TKA: Total knee arthroplasty
VAS: Visual analog scale
WOMAC: Western Ontario and McMaster Universities Osteoarthritis Index
